# Interstitial lung disease constitutes a significant treatment burden in patients with rheumatoid arthritis

**DOI:** 10.3389/fmed.2026.1756931

**Published:** 2026-04-09

**Authors:** Toshiyuki Aramaki, Kanako Kojima, Toru Michituji, Ayuko Takatani, Kaoru Terada, Katsumi Eguchi, Yukitaka Ueki, Naoki Iwamoto, Atsushi Kawakami

**Affiliations:** 1 Rheumatic Disease Centre, Sasebo Chuo Hospital, Sasebo, Japan; 2 Division of Advanced Preventive Medical Sciences, Department of Immunology and Rheumatology, Nagasaki University Graduate School of Biomedical Sciences, Nagasaki, Japan

**Keywords:** clinical disease activity index, glucocorticoid, RA associated interstitial lung disease, RA-ILD, rheumatoid arthritis, treatment to target

## Abstract

**Introduction:**

This study investigated the impact of interstitial lung disease (ILD), a prevalent complication of rheumatoid arthritis (RA), on the achievement of treatment goals in clinical practice under the treat-to-target (T2T) strategy.

**Methods:**

This retrospective observational study included patients with newly diagnosed RA who underwent chest computed tomography (CT) within 1 year of RA onset between 2016 and 2022. The presence of ILD was assessed using chest CT imaging. Treatment goals were evaluated at 6, 12, and 24 months after treatment started. The goals were low disease activity (CDAI < 10), low inflammation (CRP < 0.5 mg/dL), and glucocorticoid (GC)-free status. Then, univariate and multivariate analyses were performed to identify factors impacting goal achievement.

**Results and discussion:**

Of the 254 patients, 57 (22.4%) had RA-ILD. Patients with ILD were older, had higher Anti-citrullinated protein antibodies (ACPA) and rheumatoid factor (RF) positivity, used GCs more frequently, and had lower methotrexate usage. At all-time points, patients with ILD were significantly less likely to achieve treatment goals than those without ILD (21.2% vs. 37.8% at 6 months, *p* < 0.05; 25.0% vs. 48.9% at 12 months, *p* < 0.05; and 21.3% vs. 56.8% at 24 months, *p* < 0.01). Multivariate analysis showed that ILD, high baseline CRP, and GC use at the start of treatment were independently and negatively associated with treatment goals, with the adverse impact of ILD increasing over time. In conclusion, RA-ILD acts as a substantial barrier to the effective implementation of T2T strategies in RA.

## Introduction

Rheumatoid arthritis (RA) is a chronic, systemic inflammatory disease that should be treated to prevent joint damage. Treatment to target (T2T) by measuring disease activity and adjusting therapy accordingly optimizes outcomes in RA (1). Monitoring should be frequent in active disease (every 1–3 months) and if there is no improvement by at most 3 months after the start of treatment or the target has not been reached by 6 months, therapy should be adjusted.

According to European League Against Rheumatism (EULAR) recommendations (2), after RA diagnosis, methotrexate (MTX) should be considered first in Phase I. For patients contraindicated for MTX (or with early intolerance), other conventional synthetic DMARD (csDMARD) should be considered, and if treatment targets are not achieved within 6 months, the next treatment phase (Phase II) should be initiated. If treatment targets are not achieved in Phase II, progression to Phase III is recommended.

Despite following treatment guidelines, a considerable proportion of RA patients continue to experience residual symptoms. EULAR has proposed a definition for difficult-to-treat RA (D2TRA), which is intended for patients with rheumatoid arthritis who have progressed to Phase III or beyond. This definition assumes treatment failure with two or more biologic or targeted synthetic DMARD (b/tsDMARDs). However, in daily clinical practice, there are a considerable proportion of patients who attempt to follow the T2T strategy but are unable to carry it through for various reasons, resulting in persistent symptoms or the concomitant use of glucocorticoids (GCs) for a long period of time.

RA-related interstitial lung disease (RA-ILD) is an important organ complication of RA that can hinder therapeutic progress (3, 4). RA-ILD is said to occur in 20–30% of all RA patients (5), but the relationship between RA-ILD and the T2T strategy remains uncertain, since longitudinal evidence is very limited. The objective of this study is to explore the impact of ILD on the achievement of treatment goals in RA patients in clinical practice.

## Materials and methods

### Study design and participants

This study is a retrospective observational study. Data on patients with RA were obtained from a single-center cohort database, the SUNSET registry, which is a point-of-care observational registry database of patients with RA that provides real-world data from clinical practice, with ongoing recruitment and follow up (6). It captures patient- and disease-specific information collected during routine consultations.

This study focuses on newly diagnosed RA patients who visited the Rheumatic Disease Centre at Sasebo Central Hospital between 2016 and 2022 and underwent a chest CT scan within 1 year before or after the onset of RA. All RA patients fulfilled the 2010 ACR / EULAR RA classification criteria. The study excluded patients with severe non-respiratory comorbidities, such as chronic kidney disease, vascular disease, Alzheimer’s disease, and malignancies.

The following were extracted from the patients’ medical records: characteristics; disease activity [clinical disease activity index (CDAI)]; acute-phase reactants, such as C-reactive protein (CRP); concomitant glucocorticoid (GC) use and dosage; and complications. The duration of treatment with each drug and the reason for discontinuation were also investigated.

### Treatment goals

RA treatment was administered according to clinical guidelines and shared decision-making aimed at achieving clinical treatment targets (1) through EULAR recommendations for the management of rheumatoid arthritis with synthetic and biological disease-modifying antirheumatic drugs: 2022 update (2). Accordingly, the patients in Phase I were defined as those initially treated with csDMARD (usually MTX, but alternatives such as leflunomide or sulfasalazine if MTX was contraindicated or not tolerated). Phase II patients were defined as those who received another csDMARD (without poor prognostic factors) or a bDMARD/JAK inhibitor (with poor prognostic factors). Phase III patients were defined as those who switched to another bDMARD (same or different class) or a tsDMARD.

As this was a real-world clinical study, some RA patients who did not achieve the treatment target but for whom physicians were unable to change medication (e.g., due to comorbidities or safety concerns) were also included in the corresponding phase. We also evaluated the percentage of D2TRA during the treatment period in accordance with the EULAR definition (7).

In the present study, we aimed to examine whether ILD constitutes a burden in the therapeutic choices for RA. Standard T2T strategies use composite measures to evaluate disease activity. However, residual systemic inflammation, as indicated by high CRP levels, is also a prognostic factor for functional disability, and glucocorticoids (GCs) carry an increased risk of adverse events. Therefore, this study concluded that treatment targets cannot only focus on controlling inflammation, but also on discontinuing GC. Previous reports have demonstrated that patients with RA-ILD have significantly higher CRP levels compared with those without ILD, and that an elevated CRP such as higher than 0.5 or 1.0 mg/dL serves as a predictive factor for the development of RA-ILD (8). On the other hand, GCs are often the initial treatment in RA-ILD, and it has also been reported that the concomitant use of GCs is more frequent in patients with D2TRA compared with non-D2TRA (9, 10). Therefore, in this study, we defined our treatment target not only as low disease activity according to composite measures, but also by including CRP < 0.5 mg/dL and GC-free status. Accordingly, the comprehensive treatment goal was to achieve all of the following: low disease activity (CDAI < 10), low CRP (CRP < 0.5 mg/dL), and GC-free status. We examined how many patients achieved these at 6, 12 and 24 months after starting treatment, and to identify the barriers to achieving these goals. The allowable time window for each observation point was 1 month before and after.

### RA-ILD on chest CT findings

Interstitial shadows on chest CT scans are defined as ground glass opacities (GGO), reticulation, honeycombing, and consolidation (11). Each patient’s HRCT scans were read by two expert radiologists in clinical practice. For this study, an expert rheumatologist reviewed these images and calculated the proportion of interstitial shadow area to whole lung parenchyma in reference to the previous report of CTD-ILD (12, 13). The films were not anonymized in this study.

### Statistical analysis

Univariate analysis for between-group comparisons was conducted using the Wilcoxon rank sum test (with continuity correction) for numerical values, and the chi-squared test or Fisher’s exact test for proportions. Multivariable logistic regression analyses were performed to evaluate the association between achievement of treatment targets at 6, 12, and 24 months (dependent variables) and potential explanatory variables. Candidate independent variables included sex, age at RA onset, ACPA positivity, RF positivity, CDAI > 10, CRP > 0.5 mg/dL, concomitant GC use, concomitant MTX use, smoking status, and ILD, which were selected a priori based on clinical relevance and univariate analyses.

A stepwise variable selection procedure based on *p*-values was applied to identify independent factors associated with the outcomes. Variables with a *p*-value < 0.05 were considered statistically significant. Analyses were conducted using EZR software (14), a graphical user interface for R (version 4.5.1, The R Foundation for Statistical Computing, Vienna, Austria).

### Ethics

This study was approved by the Sasebo Chuo Hospital Human Research Ethics Committee (SCHEC 2022-04), and subject informed consent was obtained according to the principles of the Declaration of Helsinki.

## Results

### Characteristics of participants

**FIGURE 1:**
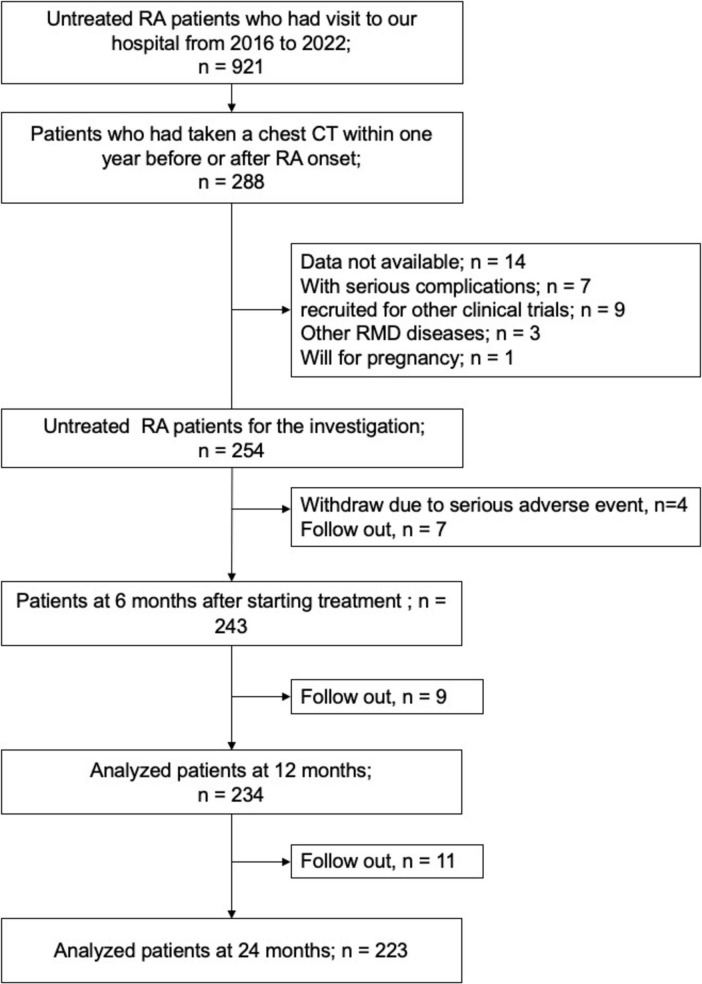
Flowchart showing patient enrollment and the number of cases analyzed at each time point. Of the 921 patients newly diagnosed with rheumatoid arthritis (RA), 288 who underwent chest 10 computed tomography (CT) within one year before or after the onset of RA were identified, and 254 11 were included in the analysis. Data were analyzed for these patients at the following time points: 12 baseline (*n* = 254), 6 months (*n* = 243), 12 months (*n* = 234) and 24 months (*n* = 223). 13 RA, rheumatoid arthritis; CT, computed tomography; RMD, rheumatic musculoskeletal disease.

Figure 1 shows the patient enrollment flowchart for the present study. Between 2016 and 2022, 288 out of 921 patients (31.2%) underwent a chest CT scan within 1 year of RA onset. Reasons for performing CT scans included assessing the risk of adverse events when transitioning to phase II therapy, and the presence of abnormalities detected through physical examination or chest X-ray. A total of 254 consecutive patients newly diagnosed with RA were evaluated in this study. As shown in Figure 1, data were analyzed from 243 patients at 6 months, 234 patients at 12 months, and 223 patients at 24 months. The objects were 254 patients, 68 of whom were male and 186 of whom were female (Table 1). The mean age at RA onset was 63.2 years. The positive rates of ACPA and RF were 63.6 and 69.8%, respectively, and the mean RF titer was 131.5 IU/ml. The mean CDAI score was 19.3. Ninety-eight patients (38.6%) had a history of smoking. Fifty-seven patients (22.4%) had interstitial lung disease (ILD), of whom 21 (8.3%) were diagnosed with ILD before the onset of RA, representing 36.8% of RA-ILD cases. The number of RA patients with interstitial shadowing in the lung parenchyma accounting for more than 5, 10, or 15% of the lung field is 44 (17.3), 33 (13.0), or 21 (8.3%), respectively. No patients were treated with home oxygen therapy (HOT) at the onset of RA. Compared with patients without ILD, those with RA-ILD were older and had a higher ACPA and RF positivity rate (see Table 1). At the start of phase I therapy, glucocorticoids (GCs) were used concomitantly in 63.2% of patients with RA-ILD, with a mean dose of 6.6 mg, which was significantly higher than in the group without ILD. MTX was used in 54.4% of patients with ILD compared with 90.9% without ILD (*p* < 0.001). The other csDMARD used in RA-ILD patients were salazosulfapyridine (SASP) at 40.6, and iguratimod (IGT) at 3.5%. No RA-ILD patients were treated with tacrolimus in Phase I.

**TABLE 1:** Baseline characteristics of RA patients.

Baseline characteristics and treatment	Total (*n* = 254)	With ILD (*n* = 57)	Without ILD (*n* = 197)	*p*-value^†^
Sex, Male/female, male (%)	68 / 186 (26.8)	19 / 38 (33.3)	50 / 141 (24.9)	0.20
Age at RA onset, years, mean (SD)	63.2 (14.0)	71.0 (9.4)	60.9 (14.4)	< 0.01
ACPA positivity, %	63.6	75.5	60.1	< 0.05
RF positivity, %	69.8	81.8	66.5	< 0.05
RF titer, U/ml (SD)	131.5 (231.8)	222.9 (422.2)	103.2 (122.6)	0.060
CDAI, mean (SD)	19.3 (11.0)	19.2 (10.5)	19.4 (11.2)	0.87
CRP, mg/dL (SD)	2.2 (3.2)	2.7 (4.1)	2.1 (2.9)	0.32
Ever smokier, patient number (%)	98 (38.6)	22 (38.6)	76 (38.3)	0.99
The proportion of the interstitial shadow area in whole lung parenchyma, > 0% patient number (%) > 5%, patient number (%) > 10%, patient number (%) > 15%, patient number (%)	57 (22.4) 44 (17.3) 33 (13.0) 21 (8.3)	57 (100) 44 (77.2) 33 (57.9) 21 (36.8)	0	–
ILD before RA onset, patient number (%)	21 (8.3)	21 (36.8)	0	–
GC concomitant, patient number (%)	118 (46.5)	36 (63.2)	82 (41.6)	< 0.01
PSL dosage, mg/day (SD)	6.1 (3.2)	6.6 (4.6)	5.9 (2.4)	0.82
MTX usage, patient number (%)	210 (82.7)	31 (54.4)	174 (90.9)	< 0.001
SASP, patient number (%) IGT, patient number (%) TAC, patient number (%)	34 (16.5) 2 (1.0) 1 (0.5)	23 (40.4) 2 (3.5) 0	11 (7.4) 0 1 (0.7)	<0.001

RA, Rheumatoid arthritis; ILD, interstitial lung disease; ACPA, anti-CCP antibody; RF, rheumatoid factor; CDAI, clinical disease activity index; CRP, C-reactive protein; GC, glucocorticoid; PSL prednisolone; MTX, methotrexate, DMARD, disease modifying anti-rheumatic drug; SASP, salazosulfapyridine; IGT, iguratimod; TAC, tacrolimus.

†Comparison between RA patients with Interstitial lung disease and those without this complication, n.s., not significant

### Treatment progress and percentage of patients achieving treatment goals

At the 12- and 24-month time points, there was no difference in the proportion of patients who progressed to Phase II or beyond between the two groups, nor in the mechanism of action of b/tsDMARDs except at 24-month (see Table 2). There was no significant difference between the two groups in the overall adoption rates of biologic agents and JAK inhibitors (data not shown). At these two time points, the rate of methotrexate use was significantly lower in patients with ILD than in those without. Furthermore, there was no significant difference in the proportion of patients who had become D2TRA, as defined in this study, by 24 months.

**TABLE 2:** Treatment phase, drugs and proportion of RA patients achieving treatment goals at 6, 12, and 24 months after starting RA treatment.

At 6 months starting RA treatment	With ILD (*n* = 55)	Without ILD (*n* = 188)	*p*-value
Phase of treatment			
I, patient number (%) II, patient number (%) III, patient number (%)	46 (83.6) 8 (14.5) 1 (1.8)	144 (76.6) 41 (21.8) 3 (1.8)	0.42
MTX concomitant, patient number (%)	29 (54.7)	164 (87.7)	< 0.001
Mode of drug action of b/tsDMARD			
TNF inhibitor, patient number (%) IL-6 inhibitor, patient number (%) Abatacept, patient number (%) JAK inhibitor, patient number (%)	3 (33.3) 3 (33.3) 2 (22.2) 1 (11.1)	15 (34.1) 20 (45.5) 4 (9.1) 5 (11.4)	0.71
a. GC free, patient number (%)	26 (47.3)	114 (60.6)	0.077
b. CDAI ≤ 10, patient number (%)	28 (50.9)	130 (69.1)	< 0.05
c. CRP < 0.5 mg/dL, patient number (%)	33 (63.5)	145 (78.0)	< 0.05
All of a, b and c, patient number (%)	11 (21.2)	65 (37.8)	< 0.05
At 12 months starting RA treatment	With ILD (*n* = 52)	Without ILD (*n* = 182)	*p*-value
Phase of treatment			
I, patient number (%) II, patient number (%) III, patient number (%)	34 (65.4) 15 (28.8) 3 (5.8)	111 (61.0) 66 (36.3) 5 (2.7)	0.56
MTX concomitant, patient number (%)	28 (65.1)	153 (90.5)	< 0.01
MoA of b/tsDMARD using at the 12th month			
TNF inhibitors, patient number (%) IL-6 inhibitors patient number (%) Abatacept patient number (%) JAK inhibitors patient number (%)	2 (18.2) 4 (36.4) 3 (27.3) 2 (18.2)	12 (36.4) 14 (42.4) 2 (6.1) 5 (15.2)	0.27
a. GC free, patient number (%)	32 (61.5)	136 (74.7)	0.062
b. CDAI ≤ 10, patient number (%)	28 (53.8)	130 (71.4)	< 0.05
c. CRP < 0.5mg/dL, patient number (%)	37 (71.2)	157 (86.3)	< 0.05
All of a, b and c, patient number (%)	13 (25.0)	89 (48.9)	< 0.01
At 24 months starting RA treatment	With ILD (*n* = 47)	Without ILD (*n* = 176)	*p*-value
Phase of treatment			
I, patient number (%) II, patient number (%) III, patient number (%)	26 (55.3) 17 (36.2) 4 (8.5)	99 (56.2) 62 (35.2) 15 (8.5)	0.99
MTX concomitant, patient number (%)	25 (53.2)	133 (75.6)	< 0.01
MoA of b/tsDMARD using at the 24th month			
TNF inhibitors, patient number (%) IL-6 inhibitors patient number (%) Abatacept patient number (%) JAK inhibitors patient number (%)	5 (23.8) 6 (28.6) 6 (28.6) 4 (19.0)	31 (40.3) 32 (41.6) 5 (6.5) 9 (11.7)	< 0.05
a. GC free, patient number (%)	32 (68.1)	157 (89.2)	< 0.01
b. CDAI ≤ 10, patient number (%)	30 (63.8)	135 (76.7)	< 0.01
c. CRP < 0.5mg/dL, patient number (%)	32 (68.1)	147 (83.5)	< 0.05
All of a, b and c, patient number (%)	10 (21.3)	100 (56.8)	< 0.01
Progression to D2TRA within 24 months, patient number (%)	3 (6.4)	9 (5.1)	0.73

RA, Rheumatoid arthritis; ILD, interstitial lung disease; ACPA, anti-CCP antibody; RF, rheumatoid factor; MoA, mode of drug action; b/tsDMARD, biologic-/targeted synthetic drug modifying anti-rheumatic drug; TNFi, TNF inhibitors; IL-6i, IL-6 inhibitors; ABT, abatacept; JAKi, JAK inhibitors; MTX, methotrexate; CDAI, clinical disease activity index; CRP, C-reactive protein; GC, glucocorticoid; D2TRA, difficult to treat RA. D2TRA is defined as meeting both of the following criteria: (1) treatment based on EULAR recommendations has been administered and treatment with two or more b/tsDMARDs with different mechanisms of action has failed; and (2) signs suggestive of active/progressive disease are present. These signs are defined as the presence of at least one of the following: moderate or high disease activity (CDAI > 10); findings and/or symptoms suggestive of active disease (CRP ≥ 0.5 mg/dL); or an inability to reduce glucocorticoid therapy (prednisone < 7.5 mg/day).

**FIGURE 2:**
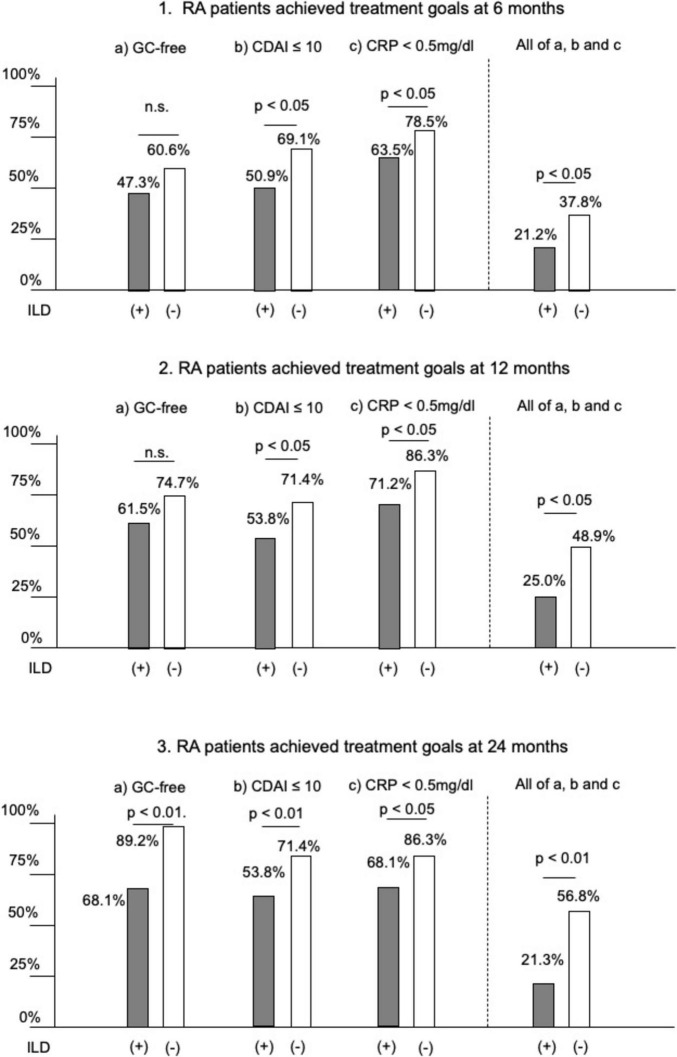
Comparison of the proportion of patients with rheumatoid arthritis who achieved the treatment goals at 6, 12, and 24 months in two groups (ILD group and no ILD group). **(1–3)** The proportion of patients who achieved the treatment goals at 6, 12, and 24 months after the start of treatment. RA, rheumatoid arthritis; ILD, interstitial lung disease; GC, glucocorticoid

The proportion of patients who achieved the treatment goals of being GC-free, CDAI ≤ 10, and CRP < 0.5 mg/dL was consistently lower in ILD patients compared with those without ILD, with the difference becoming more pronounced over time (Figure 2). At 6 months, 21.2% of ILD patients reached the treatment goals versus 37.8% of patients without ILD (*p* < 0.05). At 12 months, the respective rates were 25.0 and 48.9% (*p* < 0.05). By 24 months, the disparity had further widened, with only 21.3% of ILD patients achieving the goals compared with 56.8% of patients without ILD (*p* < 0.01).

### Factors to inhibit the achievement of treatment goals

Multivariate analysis identified several factors negatively associated with the achievement of treatment goals (Table 3). ACPA positivity was significantly associated with a reduced likelihood of reaching the treatment goals at 6 months. In addition, high baseline CRP levels ( ≥ 0.5 mg/dL) and the use of glucocorticoids at treatment initiation were consistently associated with a lower probability of achieving the goals at 6, 12, and 24 months. The presence of ILD was also negatively associated with treatment goal achievement at 12 months [odds ratio (OR) 0.41, 95% confidence interval (CI) 0.19–0.85, *p* < 0.05], and its adverse effect became even more evident at 24 months (OR 0.21, 95% CI 0.09–0.48, *p* < 0.05), indicating that the negative impact of ILD on treatment outcomes increased over time.

**TABLE 3:** Univariate and multivariate analyses for associations between baseline characteristics and treatment goals at 6-, 12-, and 24 months after starting treatment.

Treatment goal			GC-free, CDAI ≤ 10 and CRP < 0.5 mg/dl											
			At 6 months				At 12 months				At 24 months			
Baseline characteristics			Univariate		Multivariate		Univariate		Multivariate		Univariate		Multivariate	
		Ref.	OR (95%CI)	*p*-value	OR (95%CI)	*p*-value	OR (95%CI)	*p* -value	OR (95%CI)	*p*-value	OR (95%CI)	p -value	OR (95%CI)	p -value
Sex	Male	Female	0.43 (0.19–0.89)	0.015			0.80 (0.42–1.54)	0.49			1.12 (0.58 –2.17)	0.72		
Age at RA onset	≥ 65	< 65	1.24 (0.70–2.22)	0.43			0.71 (0.41–1.24)	0.20			0.529 (0.298–0.930)	0.018		
ACPA	Positive	Negative	0.64 (0.33–1.21)	0.13	0.48 (0.24–0.96)	0.038	0.897 (0.49–1.66)	0.71			1.097 (0.59–2.05)	0.75		
RF	Positive	Negative	0.97 (0.52–1.85)	0.93			1.04 (0.57–1.91)	0.89			1.657 (0.893–3.096)	0.085		
CDAI	> 10	≤ 10	0.44 (0.082 –2.45)	0.24			0.57 (0.081–3.46)	0.47			1.60 (0.26 -11.2)	0.54		
CRP	≥0.5 mg/dL	< 0.5 mg/dL	0.47 (0.26–0.849)	< 0.01	0.51 (0.26–0.99)	0.047	0.40 (0.22–0.73)	< 0.01	0.43 (0.24–0.77)	< 0.01	0.520 (0.282–0.946)	0.022	0.543 (0.30–0.99)	< 0.05
GC	> 0 mg/dL, PSL equivalent	0	0.11 (0.053–0.24)	< 0.001	0.13 (0.061– 0.27)	< 0.001	0.264 (0.14–0.471)	< 0.01	0.31 (0.17–0.54)	< 0.01	0.518 (0.292–0.911)	0.014	0.561 (0.32–0.99)	<0.05
MTX	> 0 mg/week	0	0.79 (0.37–1.71)	0.50			1.00 (0.47–2.14)	1.00			1.597 (0.734–3.52)	0.20		
Smoking	Ever	Never	0.58 (0.31–1.07)	0.065			1.20 (0.68–2.13)	0.49			1.16 (0.65–2.09)	0.58		
ILD	>0 %	0	0.47 (0.21–1.01)	0.040			0.35 (0.16–0.72)	<0.01	0.41 (0.19–0.85)	<0.05	0.28 (0.13–0.57)	<0.01	0.21 (0.09–0.48)	< 0.01
Proceed to the second or later phase	Yes	No	0.61 (0.32–1.13)	0.094			1.02 (0.58–1.79)	0.96			0.64 (0.36–1.12)	0.094		

GC, glucocorticoid; CDAI, clinical disease activity index, CRP, C-reactive protein, RA, Rheumatoid arthritis; ACPA, anti-CCP antibody; RF, rheumatoid factor; MTX, methotrexate; ILD, interstitial lung disease.

## Discussion

In this study, patients with RA-ILD were found to be older and to have higher rates of ACPA and RF positivity and glucocorticoid (GC) use, but lower rates of MTX use, at the initiation of RA treatment compared with patients without RA-ILD. ACPA positivity had a transient negative impact on achieving treatment goals under the T2T strategy at 6 months, but this effect was not observed at 12 or 24 months. In contrast, high baseline CDAI and CRP levels were consistently associated with poor outcomes across all time points. The most notable finding was that ILD comorbidity exerted a progressively negative influence on treatment goal achievement at 12 and 24 months, with its impact becoming increasingly pronounced over time.

Previous studies have reported that RF positivity tends to be lower in late-onset RA, whereas RA-ILD is characterized by elevated RF positivity rates, higher RF titers, and increased ACPA positivity (15–17). In our study as well, despite the older age of the RA-ILD group. Given that RF has been identified as a predictor of D2TRA (18), RA-ILD may further increase the risk of treatment resistance. Importantly, comorbid ILD may be overlooked in clinical decision-making due to the traditional emphasis on joint manifestations in RA (19). Our results underscore the necessity of considering pulmonary involvement when implementing T2T strategies.

From a therapeutic perspective, the frequent use of GCs in RA-ILD remains problematic. Although GCs may be effective in certain ILD patterns such as NSIP and OP (20, 21), they are less effective in UIP and may accelerate fibrosis. Moreover, GC use is associated with increased risk of adverse events, particularly infections. Thus, long-term GC use in RA-ILD patients is likely to contribute to both pulmonary and systemic complications.

In this study, the high prevalence of persistent moderate to high disease activity and ongoing inflammation in RA-ILD patients over 6–24 months appeared to be related, at least in part, to low MTX usage rates. Although some studies have suggested that MTX does not increase the risk of RA-ILD (22), the concern regarding MTX-induced pneumonitis (23–26) often leads to limited use of MTX in RA-ILD patients, thereby weakening the effectiveness of T2T strategies.

Although the proportion of patients progressing to Phase II/III or meeting the EULAR definition of D2TRA within 2 years did not differ significantly between RA-ILD and non-ILD patients, a greater proportion of RA-ILD patients failed to achieve treatment goals. Persistent inflammation and comorbid conditions, such as ILD, are well-recognized risk factors for D2TRA. However, these comorbidities are frequently underappreciated due to the predominant focus on joint manifestations in RA (1). Our findings suggest that RA-ILD itself may impair the implementation of T2T strategies, even in patients who do not fulfill the criteria for D2TRA. Moreover, because high disease activity is a risk factor for ILD progression (27–29), the persistence of systemic inflammation in RA-ILD patients not only drives joint damage but may also accelerate ILD progression, contributing to increased mortality in RA (13).

The efficacy and safety of b/tsDMARDs in RA-ILD remain heterogeneous. While TNF inhibitors have been linked to ILD progression in some studies (30, 31), they may also have antifibrotic properties (32). IL-6 inhibitors have demonstrated relative safety in this population (33, 34). Abatacept is widely used in RA-ILD due to its favorable safety profile, with meta-analyses showing a significant reduction in ILD progression compared with TNF inhibitors (35). JAK signaling, particularly JAK2/STAT3, has been implicated in the pathogenesis of RA-ILD (36–39), suggesting a mechanistic rationale for the use of JAK inhibitors. Indeed, meta-analyses have indicated that tofacitinib may carry a lower incidence of ILD among bDMARDs, even after adjustment for relevant covariates (40), making it a potential therapeutic option.

This study has several limitations. First, because this study was a retrospective observational study, there is an inherent risk of selection bias. This selection bias, particularly with regard to CT scans, may have influenced the prevalence of RA-ILD. However, as medical radiation exposure was a major concern and only tests necessary for treatment were performed in daily practice, we believe this selection bias is acceptable. Secondly, histological and functional evaluations of ILD were not conducted. Thirdly, this study did not analyze RA-ILD according to the classification of radiological patterns, such as UIP, NSIP or OP. Therefore, it remains unclear which pattern most significantly influences the treatment goals. Furthermore, no longitudinal investigation was conducted and the effect of treatment on RA-ILD progression was not examined. Fourthly, the use of a stepwise variable selection procedure may result in overfitting and unstable variable selection depending on the chosen *p*-value thresholds, and the identified associations should therefore be interpreted with caution. Nevertheless, despite these limitations, our study highlights the significant impact of RA-ILD on T2T strategies in clinical practice and contributes valuable insights into this underexplored area.

Importantly, the negative impact of ILD on treatment outcomes increased over time, suggesting that RA-ILD acts as a substantial barrier to the effective implementation of T2T strategies in RA. In daily clinical practice, clinicians may be reluctant to treat patients with RA-ILD due to the risk of acute exacerbation and safety concerns. This may also explain why ILD hinders the achievement of T2T treatment goals. Careful balancing of GC use, cautious consideration of MTX, and preferential use of biologics with favorable pulmonary safety profiles should be prioritized. Furthermore, given that persistent inflammation accelerates both joint damage and ILD progression, aggressive but tailored disease control is essential to improve long-term outcomes and survival in patients with RA-ILD.
